# *MYOC* and *FOXC1* gene analysis in primary congenital glaucoma

**Published:** 2010-10-08

**Authors:** Mukesh Tanwar, Manoj Kumar, Tanuj Dada, Ramanjit Sihota, Rima Dada

**Affiliations:** 1 Laboratory For Molecular Reproduction and Genetics, Department of Anatomy, All India Institute of Medical Sciences, Ansari Nagar, New Delhi, India; 2 Dr. R.P. Centre for Ophthalmic Sciences, All India Institute of Medical Sciences, Ansari Nagar, New Delhi, India

## Abstract

**Purpose:**

To screen the myocilin (*MYOC*) and forkhead box protein C1 (*FOXC1*) genes for sequence variations in primary congenital glaucoma (PCG).

**Methods:**

Seventy five PCG patients were screened for *MYOC* variations and 54 cases (negative or heterozygous for cytochrome P4501B1 mutations) for *FOXC1* mutations by polymerase chain reaction (PCR) and DNA sequencing.

**Results:**

Five single nucleotide polymorphisms (SNPs; −126T>C, −83G>A, p.R76K, IVS2+35G>A, and p.Y347Y) were identified in *MYOC* and two sequence variations (GGC375ins and GGC447ins) in *FOXC1*. No pathogenic variations were identified in *MYOC* and *FOXC1* in our patients.

**Conclusions:**

*MYOC* and *FOXC1* mutations are not involved in pathogenesis of primary congenital glaucoma in our patients. Thus, it is important to screen other loci for involvement in congenital glaucoma in cases which are negative or heterozygous for *CYP1B1* mutations to have a better insight in to disease pathogenesis.

## Introduction

Glaucoma is an optic neuropathy, characterized by elevated intra-ocular pressure (IOP) which results in retinal ganglion cell (RGC) death and vision loss [1]. Primary congenital glaucoma (PCG; OMIM 231300) is a severe form of glaucoma which manifests in the neonatal/early infantile period with a classic triad of symptoms viz. epiphora (excessive tearing), photophobia (hypersensitivity to light), and blepharospasm [2]. The prevalence of congenital glaucoma varies across ethnic communities and geographical boundaries [3]. In the Indian state of Andhra Pradesh, its prevalence is estimated to be around 1:3,300 and accounts for 4.2% of all cases of childhood blindness [4].

Genetic heterogeneity is the hallmark of PCG and three chromosomal loci (I) 2p21 (GLC3A (GLC stands for glaucoma); OMIM 231300) [5], (II) 1p36 (GLC3B; OMIM 600975) [6], and (III) 14q24.3 (GLC3C) [7] have been mapped by linkage analysis, of which only the GLC3A locus harboring the human Cytochrome P450 gene (*CYP1B1*; OMIM 601771) has been characterized [8]. *CYP1B1* exhibits a high degree of allelic heterogeneity and more than 79 different mutations associated with PCG have been identified [9]. The proportion of PCG cases with *CYP1B1* mutations vary widely across different populations and are highest among the inbred Slovakian Gypsy [10] and Saudi Arabian populations [11] which exhibit allelic homogeneity.

Earlier we reported *CYP1B1* mutations as a predominant cause for PCG phenotype [12,13]. We observed *CYP1B1* mutations in 42.66% PCG cases (32/75; 13 cases were homozygous, 8 were compound heterozygous, and 11 were heterozygous for *CYP1B1* mutations). However, 57.34% of these cases did not show the involvement of *CYP1B1*.

This led us to explore the role of other genes to understand their possible implications in the disease pathogenesis. The myocilin gene implicated in juvenile open angle glaucoma (JOAG) and in adult-onset primary open angle glaucoma (POAG) [14] was chosen as the potential candidate for screening these cases. The myocilin gene (*MYOC*) exhibits a wide spectrum of mutations and accounts for 2–5% cases of POAG [15] and in 5.5% of PCG [16]. *MYOC* is located on chromosome 1 at 1q25 and codes for myocilin/trabecular meshwork-induced glucocorticoid response (TIGR) protein. Most tissues of the eye express MYOC, including trabecular meshwork, sclera, ciliary body, and retina [17,18]. An earlier report showed that MYOC interacts with CYP1B1 through a digenic mechanism in causing JOAG [19].

Forkhead box protein C1 (*FOXC1/FKHL7*), another gene, was also selected for mutation analysis in PCG cases which were either negative or heterozygous for *CYP1B1* mutations. A recent study from Southern India has reported involvement of *FOXC1* mutations in PCG [20]. *FOXC1* is a member of the winged helix/forkhead family of transcription factors. It is located on 6p25 and has single exon that codes for 553 amino acids long protein [21]. The FOXC1 protein is expressed in various ocular and non-ocular tissues [22,23] and is found in periocular mesenchyme cells that give rise to ocular drainage structures such as the iris, cornea, and trabecular meshwork [24]. Both the *FOXC1* null (*FOXC1*^−/−^) and the heterozygous (*FOXC1*^+/−^) mice were found to have anterior segment abnormalities similar to those in humans with anterior segment dysgenesis (ASD) and congenital glaucoma [25]. In this study we have screened *MYOC* gene in 75 PCG cases and *FOXC1* gene in 54 PCG cases (negative/heterozygous for *CYP1B1* mutations) to understand the role of these two gene in the pathogenesis of this blinding disorder.

## Methods

Congenital glaucoma cases presenting at the Dr. Rajendra Prasad Centre for Ophthalmic Sciences, All India Institute of Medical Sciences (AIIMS), New Delhi, India, were enrolled for this study. After ethical approval of the Institutional Review Board (IRB00006862; AIIMS) a total of 75 PCG cases were screened for *MYOC* sequence variations while *FOXC1* was screened in 54 cases (either negative or heterozygous for *CYP1B1* mutations; Table 1). The diagnosis involved clinical, ocular and systemic examination. Inclusion criteria of the patients were increased corneal diameter (>12.0 mmHg) and raised IOP (>21 mmHg) with presence/absence of Haab’s striae and optic disc changes (where examination was possible). Symptoms of epiphora and photophobia were the additional inclusion factors. The age of onset ranged from birth to 3 years.

**Table 1:** Clinical phenotype and *CYP1B1* mutation status of PCG cases.

**Pt. ID**	**Age of onset of disease**	**Sex**	**Age at presentation/Sampling**	**Corneal Diameter (mm) OS/OD**	**Buphthalmos**	**IOP OS**	**IOP OD**	**Haabs’ striae**	**Last Cup Disc ratio OS/OD**	**Corneal edema**	**Mutations**	**Treatments**
PCG01	By birth	M	12 years	15x15/15x15.5	OU	40	30	OU	Total cupping	OU	R390H (H)	Medical and OU 3XTrab/Trab+MMC
PCG02	By birth	F	10 years	13x13/13x13	OU	36	40	OU	0.5:1/0.6:1	OU	R390H (H)	Medical and OU 1XTrab/Trab+MMC
PCG03	By birth	F	7 months	15x14/15x15	OU; OD>OS	26	38	Absent	Hazy MEDIA	Absent	R390H (H)	Medical and OU 1XTrab/Trab+MMC; OS 1XTrab/Trab+MMC
PCG04	By birth	M	10 months	12x11/12x12	Absent	22	24	Absent	0.4:1/0.4:1	Absent	—	Medical and OU Trab/Trab+MMC
PCG05	4 months	M	5 months	14.5x15/15x15	OU; OD>OS	28	28	OU	0.7:1/0.7:1	OU	R368H (H)	Medical and OU 2XTrab/Trab+MMC
PCG06	By birth	M	4 months	12x13/12x13	OU	22	23	OU	0.5:1/0.5:1	OU	—	Medical and OU Trab/Trab+MMC
PCG07	By birth	F	18 months	12x13/11x11.5	OS	38	14	Absent	0.4:1/0.4:1	Absent	—	Medical and OU Trab/Trab+MMC
PCG08	1 month	F		NA	OD	23	25	Absent	Hazy media	One eye	—	Medical and OD Trab/Trab+MMC
PCG09	2 months		13 months	12x12/12x12	Absent	22	22	Absent	NA	Absent	—	Medical and OU Trab/Trab+MMC
PCG010	1 month	F	1 month	NA	OD	23	24	Absent	NA	One eye	—	Medical and OD Trab/Trab+MMC
PCG011	1 year	M	4 months	14x14/14x15	OU; OD>OS	26	22	Absent	NA	Absent	E229K (h)	Medical and OU 1XTrab/Trab+MMC
PCG012	By birth	M	58 months	14x14.5/14x14.4	OU	32	32	Absent	NA	Absent	R368H (h)	Medical and OU 1XTrab/Trab+MMC
PCG013	By birth	F	2 months	14x14/14x14	OU	31	30	Absent	Hazy media	Absent	R390H (H)	Medical and OU 2XTrab/Trab+MMC
PCG014	By birth	F	1 month	NA	OU	25	24	Absent	Hazy media	Absent	E229K (h)	Medical and OU 2XTrab/Trab+MMC
PCG015	By birth	M	3 months	14.5x14/13.5x13	OU; OS>ODR	32	32	Absent	0.6:1/0.6:1	Absent	M132R (H)	Medical and OU 2XTrab/Trab+MMC
PCG016	By birth	M	6 months	11x11/12x12.5	OD	18	26	Absent	0.4:1/0.5:1	Absent	—	Medical and OD Trab/Trab+MMC
PCG017	By birth	M	9 months	14x14/14x14.5	OU	30	28	Absent	NA	Absent	Ter@223 (H)	Medical and OU 2XTrab/Trab+MMC
PCG018	By birth	M	3.4 years	14.5x14/14x14	OU; OS>OD	20	20	Absent	NA	Absent	—	Medical and OU Trab/Trab+MMC
PCG019	By birth	F	7 months	12x12.5/12x12	OU	22	22	Absent	0.5:1 1	Absent	Ter@223 (h)	Medical and OU 2XTrab/Trab+MMC
PCG020	By birth	M	7 years	12x13/13x13	OU; OD>OS	18	37	Absent	Hazy media	Absent	—	Medical and OU1X Trab/Trab+MMC
PCG021	By birth	M	2 years	15x16/11.5x12	OS	32	15	OU	Hazy media	Absent	Ter@223 (h)	Medical and 2X Trab/Trab+MMC OS
PCG022	By birth	F	10 months	15x15/16x16	OU; OD>OS	28	28	OU	0.7:1/0.5:1	Absent	p.L24R (H)	Medical and OS 2XTrab/Trab+MMC
PCG023	By birth	F	4 months	14x15/14x15	OU	34	36	OU	Hazy media	OU	—	Medical and OU 1X Trab/Trab+MMC
PCG024	By birth	M	1 months	14x13/14x13	OU	30	24	Absent	0.7:1/0.7:1	OU	—	Medical and OU 1X Trab/Trab+MMC
PCG025	By birth	F	18 years	NA	Absent	32	10	Absent	NA	Absent	—	Medical and OS Trab/Trab+MMC
PCG026	By birth	M	2 years	14x15/14x15	OU	22	22	Absent	Hazy media	OU	—	Medical and OU Trab/Trab+MMC
PCG027	By birth	F	2 years	13x13.5/15x14.5	OU;	22	22	Absent	0.8:1/0.8:1	Absent	—	Medical and OU 1X Trab/Trab+MMC
PCG028	By birth	M	2.5 years	13x13.5/13x13.5	OU	20	20	Absent	hazy media	One eye	—	Medical and OU 1X Trab/Trab+MMC
PCG029	By birth	F	3 months	15x15/14x14	OU	28	27	OU	Hazy media	OU	Ter@223 (h)	Medical and 2X Trab/Trab+MMC
PCG030	By birth	M	1 month	13x13.5/13.5x13	OU	26	26	Absent	0.7:1/0.7:1	One eye	—	Medical and 1X OU Trab/Trab+MMC
PCG031	By birth	M	4 months	11x14/14x15	OU	34	36	Absent	Hazy media	Absent	—	Medical and 1X OU Trab/Trab+MMC
PCG032	By birth	M	10 months	15x15/12x12	OS	18	14	Absent	0.3:1/0.3:1	Absent	—	Medical and 1X OS Trab/Trab+MMC
PCG033	By birth	M	1 year	13x13/11x11	OS	12	12	Absent	0.2:1/NO glow	One eye	—	Medical and 1X OS Trab/Trab+MMC
PCG034	By birth	M	2 months	14x14/14x14	OU	22	24	Absent	NA	Absent	R390H (h); Ter@223 (h	Medical and 1X OU Trab/Trab+MMC
PCG035	By birth	M	3 months	13x13/13x12	OS	18	16	Absent	no glow	Absent	—	Medical and 1X OS Trab/Trab+MMC
PCG036	By birth	M	1 month	15x15/15x15.5	OU	25	26	Absent	0.8:1 1	Absent	R390H (H); E229K (h)	Medical and 1X OD Trab/Trab+MMC
PCG037	By birth	M	1 month	14x14/11x11	OU;OS>OD	18	22	Absent	Hazy media	One eye	—	Medical and 1X OU Trab/Trab+MMC
PCG038	By birth	M	3.5 years	corneal: Dulcer/14x14	OD	18	20	Absent	hazy media	OU	—	Medical and 1X OD Trab/Trab+MMC
PCG039	By birth	M	4.5 years	13x13/12x13.5	OU	36	34	Absent	NT cupping	OU	R368H (h); Ter@223 (h)	Medical and 1X OUTrab/Trab+MMC
PCG040	By birth	F	2 months	11.5x12.5/12x13	OU; OD>OS	23	23	Absent	Hazy media	OU	Ter@223 (h)	Medical and OD 1XTrab/Trab+MMC
PCG041	By birth	M	11 months	12x10/12x12.5	OU; OD>OS	40	26	Absent	Hazy media	OU	R390H (H): Ter@223 (h)	Medical and OU 1XTrab/Trab+MMC; 1XTrab+Trab+mmc OD, 1XPK
PCG042	By birth	M	5 days	13x13/13x13	OU	22	24	Absent	Hazy media	OU	R368H (H) F190L (H)	Medical and 1XTrab/Trab+MMC OU
PCG043	By birth	F	I2 months	12x12/12x12	OU	22	26	OU	0.8:1 1	Absent	R390H (h) G329D (h)	Medical and 1X OU Trab/Trab+MMC
PCG044	By birth	M	4 years	14x14/14x14	OU	26	24	Absent	0.8:1/0.9:1	Absent	Ter@223(h)	Medical and 1X OU Trab/Trab+MMC
PCG045	By birth	M	1 month	12x12/12x12.5	OU	20	22	Absent	0.5:1/0.5:1	Absent	E229K (h), R390H (h)	Medical and 1X OU Trab/Trab+MMC
PCG046	By birth	M	6 months	13x13/13x13.5	OU	24	16	Absent	0.5:1/0.3:1	OU	—	Medical and1X OU Trab/Trab+MMC
PCG047	By birth	F	5 months	14.5x14/14x14	OU	30	20	Absent	0.7:1/0.9:1	One eye	—	Medical and 1X OU Trab/Trab+MMC
PCG048	By birth	M	11 months	13x13.5/12x13	OU	28	26	Absent	NA	Absent	—	Medical and 1X OU Trab/Trab+MMC
PCG049	By birth	M	6 months	18x20/14x16	OU	24	22	Absent	OS no glow/0.5:1	Absent	—	Medical and 1X OU Trab/Trab+MMC
PCG050	By birth	F		11x11.5/11x11.5	OU	26	30	Absent	0.3:1/0.3:1	Absent	—	Medical and 1X OU Trab/Trab+MMC
PCG051	By birth	F	36 months	11x11.5/13x13	OU;OD>OS	22	28	Absent	0.8:1/0.9:1	OU	—	Medical and 1X OU Trab/Trab+MMC; OU cataract surgery
PCG052	By Birth	F	2 months	12.5x13/12.5x13	OU	20	20	Absent	Hazy media/0.5:1	Absent	—	Medical and 1X OU Trab/Trab+MMC
PCG053	By birth	F	4 months	11.5x12/12x12	OU; OD>OS	40	23	Absent	Hazy media	OU	—	Medical and 1X OU Trab/Trab+MMC
PCG054	By birth	M	9 months	15x14.5/15x14.5	OU	26	26	Absent	Absent glow	Absent	—	Medical and 1X OU Trab/Trab+MMC
PCG055	By birth	M	8 months	Phthisic eye/12x12	OD; OS Phthisic eye	na	37	Absent	NA/0.9:1	OU	p.I94X (H)	Medical and 1X OD Trab/Trab+MMC
PCG056	By birth	F	12 months	14.5x14.5/14x14	OU; OS>OD	30	28	OU +ve	Hazy media	OU	p.Q340H (H) + p.R390H (H)	Medical and 1X OU Trab/Trab+MMC
PCG057	By birth	M	3 months	13x13/13.5x13.5	OU; OD>OS	28	30	Absent	0.7:1/0.7:1	Absent	—	Medical and 1X OU Trab/Trab+MMC
PCG058	By birth	M	15 months	14x14/12.5x12.5	OU; OS>OD	20	16	Absent	total cupping/ 0.5:1	Absent	—	Medical and 1X OU Trab/Trab+MMC
PCG059	By birth	F	10 months	14x14.5/13.5x14	OU; OS>OD	31	31	OS	NA	Absent	—	Medical and 1X OU Trab/Trab+MMC
PCG060	7 months	F	41 months	14x14/13x13.5	OU; OS>OD	24	18	Absent	0.4:1/0.6:1	Absent	—	Medical and 1X OU Trab/Trab+MMC
PCG061	By birth	M	4 months	15.5x15/14x14	OU; OS>OD	30	34	OU	Not visible/0.9:1	OU	p.H279D (h)	Medical and 1X OU Trab/Trab+MMC
PCG062	By birth	M	8 months	11x11.5/10x11.5	OU; OS>OD	22	16	Absent	0.6:1/0.6:1	Absent	—	Medical and 1X OU Trab/Trab+MMC
PCG063	3 months	M	12 months	12x12/11x12.5	OU; OS>OD	22	23	Absent	0.4:1/0.4:1	Absent	—	Medical and 1X OU Trab/Trab+MMC
PCG064	By birth	M	1 month	12x12/11.5x11.5	OU; OS>OD	28	24	Absent	not visible/0.7:1	Absent	p.R390C (h)	Medical and 1X OU Trab/Trab+MMC
PCG065	By birth	M	6 months	13x13/12.5x13	OU; OS>OD	25	26	Absent	0.7:1/0.7:1	OU	p.E229K (h)	Medical and 1X OU Trab/Trab+MMC
PCG066	3 months	M	13 months	12.5x12/12.5x12	OU	22	24	Absent	0.4:1/0.4:1	Absent	—	Medical and 1X OU Trab/Trab+MMC
PCG067	11 months	M	132 months	12x13/13x14	OU; OD>OS	21	24	Absent	not visible	OU	—	Medical and 1X OU Trab/Trab+MMC
PCG068	By birth	M	6 months	13x13/13.5x14	OU; OD>OS	26	28	Absent	0.8:1/0.8:1	OU	p.R368H (H)	Medical and 1X OU Trab/Trab+MMC
PCG069	By birth	M	4 months	13x13/12x13	OU; OS>OD	24	26	Absent	0.5:1/0.6:1	Absent	—	Medical and 1X OU Trab/Trab+MMC
PCG070	By birth	M	45 days	15x15/15x14.5	OU/; OS>OD	30	40	Not visible	not visible	OU	p.R355X (H)	Medical and 1X OU Trab/Trab+MMC
PCG071	13 months	M	18 months	12x12/12x12	OU	22	23	Absent	0.4:1/0.4:1	Absent	—	Medical and 1X OU Trab/Trab+MMC
PCG072	By birth	M	45 days	12x12/12x11.5	OU; OS>OD	20	24	Absent	no glow	OU	—	Medical and 1X OU Trab/Trab+MMC
PCG073	By birth	M	2 months	12.5x13/11x12	OU; OS>OD	22	22	Absent	Hazy media	OU	—	Medical and 1X OU Trab/Trab+MMC
PCG074	By birth	M	8 months	14x14.5/15x15	OU;OS>OD	28	24	OU	No glow	OU	p.R368 (H)	Medical and 1X OU Trab/Trab+MMC
PCG075	By birth	M	1 year	13x13.5/14x14	0U; OD>OS	20	20	Absent	No glow	OU	R390H (H)	Medical and 1X OU Trab/Trab+MMC

Key: M- male; F- female; H- homozygous; h-heterozygous; X- times; Trab/Trab+MMC- combined trabeculotomy trabeculectomy and mitomycin C treatment; OD- right eye; OS- left eye; OU- both eyes; NA- not available.

All patients with history of blood transfusion, TORCH (Toxoplasmosis; Rubella; Cytomegalovirus; Herpes Simplex Virus) infection and drug intake by mother during pregnancy were excluded. Glaucoma cases other than PCG were excluded. Detailed family history of ocular or other hereditary disorders up to three generations were taken, and pedigree charts were constructed.

### Control group

Seventy five ethnically matched normal individuals without any ocular/systemic disorders were enrolled as controls for *MYOC* and 50 for *FOXC1* analysis. Peripheral blood samples were collected from patients and controls by venipuncture in ethylenediaminetetra-acetic acid (EDTA) vacutainers only after informed consent and stored in −80 °C until further use.

### Mutation screening and sequence analysis

DNA was isolated from the peripheral blood using the Phenol chloroform method. All three exons with exon-intron boundaries were amplified from DNA using polymerase chain reaction (PCR) primers designed for *MYOC* (GenBank AB006688; available at Genbank) [26]. *FOXC1* primers (Table 2) were designed using National Center for Biotechnology Information (NCBI) PRIMER3 program. PCR amplifications for *FOXC1* primers were performed in a 40 μl volume containing 1.0 μl of 20 μM stock solution for each primer, 100 ng of genomic DNA, 1 unit of Taq polymerase (Banglore Genei P Ltd, Bengaluru, Karnataka, India), 0.1 mM of each dNTP and 4 μl of 10× PCR buffer (with 15 mM MgCl_2_), by means of 35 cycles of amplification, each consisting of 30 s denaturation at 94 °C, 50 s annealing at 56 °C −59 °C and 1 min extension at 72 °C and final extension at 72 °C for 5 min.

**Table 2:** *FOXC1* primers used in this study.

**Serial number**	**Primer sequence**	**Product size**
1	1F- CCCGGACTCGGACTCGGC	649 bp
** **	1R- TCTCCTCCTTGTCCTTCACC	** **
2	2F- GAGAACGGCAGCTTCCTG	708 bp
** **	2R-TTGCAGGTTGCAGTGGTAGGT	** **
3	3F-GGCCAGAGCTCCCTCTACA	636 bp
** **	3R-CTGCTTTGGGGTTCGATTTA	** **

All PCR products were analyzed on 1.8% agarose gel, stained with ethidium-bromide (EtBr 10 mg/ml). Agarose gel was analyzed using gel documentation system (Applied biosystems, Carlsbad, CA). Amplified PCR products were purified using gel/PCR DNA fragments extraction kit (Catalog number DF100; Geneaid Biotech Ltd., Sijhih City, Taiwan). Purified PCR product were sent for sequencing at MCLAB (Molecular Cloning Laboratories, South San Francisco, CA). DNA sequences were analyzed against the *MYOC* reference sequence and the *FOXC1* reference sequence using ClustalW2 provided by the European Molecular Biology Laboratory (EMBL) European Bioinformatics Institute (EBI).

### Computational assessment of missense mutations

Two homology based programs PolyPhen (Polymorphism Phenotyping) and SIFT (Sorting Intolerant From Tolerant) analysis tool were used to predict the functional impact of missense changes identified in this study [27,28]. The prediction is based on the position-specific independent counts (PSIC) score derived from multiple sequence alignments of observations. PolyPhen scores of >2.0 indicate the polymorphism is probably damaging to protein function. Scores of 1.5–2.0 are possibly damaging, and scores of <1.5 are likely benign. SIFT is a sequence homology-based tool that sorts intolerant from tolerant amino acid substitutions and predicts whether an amino acid substitution in a protein will have a phenotypic effect [29,30]. Positions with normalized probabilities less than 0.05 are predicted to be deleterious and, those greater than or equal to 0.05 are predicted to be tolerated. We have also used an improved splice site predictor tool [31] to predict whether a nucleotide change is likely to create a splice site.

### Statistical analysis

The frequency of nucleotide variation between cases and controls was calculated by χ^2^/Fisher’s exact test and a p-value ≤0.05 was considered significant.

## Results

A total of five nucleotide changes (2 in promoter region, 2 in coding region and 1 in intronic region) were observed in *MYOC* and two sequence variations (GGC375ins and GGC447ins) were observed in *FOXC1* in this study. Details of all these changes are given below.

### Myocilin (*MYOC*) gene

#### −126 Thymine to Cytosine (−126T>C)

**Figure 1:**
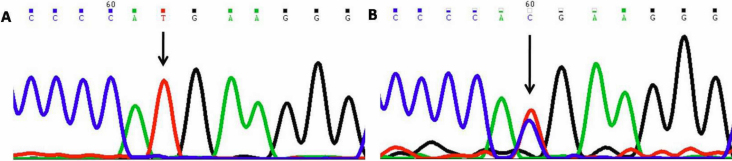
DNA sequence chromatogram of *MYOC* equivalent to bases −121 to −132. **A**: The reference sequence derived from control is shown. **B**: Sequence derived from congenital glaucoma patient PCG043 shows heterozygous −126T>C nucleotide change.

This nucleotide change resulted in thymine (T) being replaced by cytosine(C; Figure 1) at position g.169888563 or 126 base pairs upstream (−126 position). This change was homozygous in one (PCG043) and heterozygous in one case (PCG053) but was absent in controls.

#### −83 Guanine to Adenine (−83G>A)

**Figure 2:**
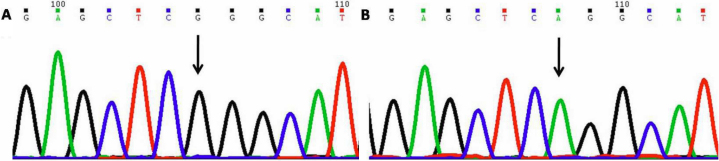
DNA sequence chromatogram of *MYOC* equivalent to bases −76 to −88. **A**: The reference sequence derived from control is shown. **B**: Sequence derived from congenital glaucoma patient PCG015 shows homozygous −83G>A nucleotide change.

This nucleotide change resulted in guanine (G) being replaced by alanine (A; Figure 2) at position g.169888457 or 83 base pairs upstream (−83 position). This change was homozygous in five cases (PCG07, 015, 034, 040, and 062) and heterozygous (PCG06, 15, 34, and 40) in four cases. This change was also present in 2 controls.

#### Arginine76Lysine (p.R76K)

**Figure 3:**
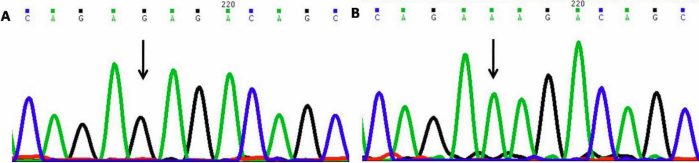
DNA sequence chromatogram of *MYOC* exon 1 equivalent to codon 75–78. **A**: The reference sequence derived from control is shown. **B**: Sequence derived from congenital glaucoma patient PCG005 shows homozygous c.127G>A, which predicts a codon change AGA>AAA and p.R76K change.

This nucleotide change resulted in G being replaced by A (Figure 3) at position g.169888148; coding nucleotide number c.127. This resulted in a codon change from AGA to AAA and amino acid change from arginine to lysine (p.R76K) a non-synonymous mutation in *MYOC*. This change was homozygous in five patients (PCG007, 015, 034, 040, and 052) and heterozygous in twenty five patients (PCG005, 006, 011, 012, 0016–019, 021, 022, 027, 033, 036, 043, 044, 046, 049, 059, 062, and 065–069) and was also present in 12 controls.

#### IVS2+35 Guanine to Adenine (IVS2+35G>A)

**Figure 4:**
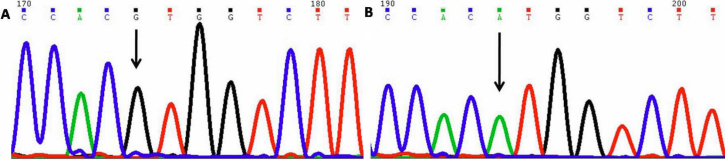
DNA sequence chromatogram of *MYOC* equivalent to IVS2+31 to IVS2+42 **A**: The reference sequence derived from control is shown. **B**: Sequence derived from congenital glaucoma patient PCG004 shows homozygous IVS2+35G>A nucleotide change.

This nucleotide change resulted in G being replaced by A (Figure 4) at position g.169874325 or 35 base pairs downstream (IVS2+35 position) ; This change was homozygous in forty seven patients (PCG002, 004, 006–009, 011–013, 015, 017, 019–022, 026–029, 033–036, 039–041, 043–046, 049–060, 063–066, and 069) and heterozygous in thirteen patients (PCG001, 003, 005, 010, 016, 018, 023, 037, 042, 054, 061, 062, and 067) and was also present in 25 controls.

#### Tyrosine347Tyrosine (p.Y347Y)

**Figure 5:**
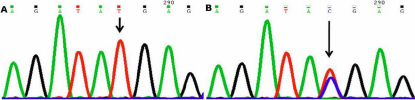
DNA sequence chromatogram of *MYOC* exon 3 equivalent to codon 346–348. **A**: The reference sequence derived from control is shown. **B**: Sequence derived from congenital glaucoma patient PCG029 shows heterozygous c.1041T>C, which predicts a codon change TAT>TAC and p.Y347Y change.

This mutation resulted in T being replaced by C (Figure 5) at position g.169872162; coding nucleotide number c.1041. This resulted in a codon change from TAT to TAC with no amino acid change (synonymous mutation) at 347 (p.Y347Y) in *MYOC* protein. Five cases (PCG029, 036, 043, 053, and 056) were heterozygous for this change while the same was absent in controls. All *MYOC* sequencing results have been tabulated (Table 3).

**Table 3:** *MYOC* gene variations identified in this study with p-value at 95% confidence interval by using Pearson χ^2^/Fisher’s exact test.

** **	** **	** **	** **	** **	**Patient ID**			** **
**Serial number**	**Location**	**Sequence change**	**Codon change**	**Mutation**	**PCG (n=75)**	**Controls (n=75)**	**p-value**	**Odds Ratio (at 95% CI)**
1	Promoter	−126T>C	-	-	2	0	0.496	-
2	Promoter	−83G>A	-	-	9	2	0.055	4.97 (1.03–23.87)
3	Exon 1	c.227 G>A	AGA>AAA	p.R76K	30	12	0.001	3.50 (1.61–7.56)
4	Intron 2	IVS2+35 G>A	-	-	60	25	<0.001	8.00 (3.80–16.80)
5	Exon 3	c.1041T>C	TAT>TAC	p.Y347Y	5	0	0.058	-

### Forkhead box protein C1 (*FOXC1*) gene

#### Insertion of GCG at g.1556820

Insertion of tri-nucleotide GCG at g.1556820 was observed in nine PCG cases (PCG006–008, 011, 023, 048, 051, 054, and 072) and four controls. This insertion was found in both alleles. This caused an insertion of an extra GGC triplet and an extra amino acid glycine at position 375 (GGC375ins). This leads to presence of seven glycine residues (generally six glycine are found) at amino acid position 375–381 instead of six glycine residues.

#### Insertion of CGG at g.1557040

Homozygous insertion of tri-nucleotide CGG at g.1557040 was observed in six PCG cases (PCG006, 007, 051, 059, 063, and 065) and five controls. This caused an insertion of an extra GGC triplet which inserts an extra amino acid glycine at amino acid position 447 (GGC447ins). This leads to presence of eleven glycine residues at amino acid position 447–457.

Three PCG cases (PCG006, 007, and 051) had both insertions (GGC375ins and GGC447ins). No other changes were identified in any PCG patient. All *FOXC1* variations have been tabulated (Table 4).

**Table 4:** *FOXC1* variations identified in this study with p-value at 95% confidence interval by using Pearson χ^2^/Fisher’s exact test.

** **	** **	** **	**Patient ID**			** **
**Serial number**	**Sequence change**	**Mutation**	**PCG (n=54)**	**Controls (n=50)**	**p-value**	**Odds ratio (at 95% CI)**
1	Ins GCC	GGC375ins	5	4	0.240	2.3 (0.66–8.00)
2	Ins CGG	GGC447ins	6	5	1	1.12 (0.32–3.94)

### SIFT and PolyPhen analysis

There was only one missense change in *MYOC* in our study. The PSIC score of the p.R76K mutation was <2 indicating that this change was benign to protein function. The SIFT score of p.R76K was 0.00 and was predicted to be deleterious for the protein function. Ideally, to call a change pathogenic both SIFT and PolyPhen results should be pathogenic. Since this change was present both in PCG and controls, it could be hypothesized that this change is non-pathogenic. Improved splice site prediction for IVS2+35G>A showed that this location (IVS2+35) is not present at splice site and may not create a splicing error in *MYOC*.

## Discussion

PCG shows marked genetic heterogenity thus we have  screened *MYOC* in 75 PCG cases to study digenic inheritance and *FOXC1* in 54 cases which were either negative or heterozygous for the *CYP1B1* mutation for their involvement. To the best of our knowledge this is the first study from north India which involves *MYOC* and *FOXC1* screening in PCG. In our study three single nucleotide polymorphisms (SNPs; Table 3) were observed both in PCG cases as well as in controls while two were limited to cases only. The frequency of p.R76K, IVS2+35G>A, and p.Y347Y in *MYOC* was found to be statistically significant (p<0.05) in our study. However, no pathogenic *MYOC* mutation was detected. A recent study showed that a small proportion of PCG cases that do not harbor *CYP1B1* mutations exhibit a heterozygous mutation in the *MYOC* gene [16]. Digenic inheritance of the mutant *MYOC* and *CYP1B1* alleles has also been demonstrated in juvenile-onset POAG and *CYP1B1* has been suggested to be a modifier of *MYOC* expression [19]. The heterozygous p.Gln48His *MYOC* mutation was first reported in two sporadic cases of JOAG and an adult-onset POAG case from eastern India. Later this change was also reported in a JOAG family and in a sporadic POAG case from India [32].

Although IVS2+35G>A was not at splice location but recent studies have revealed that intronic sequences may be associated with gene regulation. Intronic mutation, therefore, may be involved in the disease irrespective of whether the mutated base was at splice site [33]. Two promoter sequence variations (−126T>C and −83G>A) were observed in this study. These variations do not alter any known promoter or enhancer binding sites and are found in similar frequency in  patients and controls [34]. There is no evidence from literature that the −83G>A and −126T>C variations are associated with either the steroid response or POAG [34]. Further, the majority (96%) of the steroid responders examined by Fingert et al. [34] harbored no variations that met the criteria for potential involvement in the steroid response phenotype, clearly indicating that variations in the proximal promoter are not a common cause of glaucoma. All these five changes of MYOC identified in this study are listed as a neutral polymorphisms and no pathogenic variation in *MYOC* was observed in this study.

Two changes (GGC375ins and GGC447ins) in *FOXC1* were observed in cases as well as in controls. The frequency of these *FOXC1* variations were found to be statistically non-significant (p>0.05) in our study. Both of these changes have already been described in the literature as neutral polymorphisms [20,35,36]. The presence of mutations in the *FOXC1* gene in patients with PCG was initially described in an independent study conducted by Chakrabarti et al. [20] in 2009 from south India. In addition, *FOXC1* mutations have been implicated in anterior segment dysgenesis (ASD) such as iridogoniodysgenesis, Axenfeld-Rieger syndrome (ARS) and Peter’s anomaly that progress to glaucoma in 50% to 75% of cases [37-40]. Some of these ASD cases are associated with congenital or early-onset glaucomas, whereas some have glaucoma secondary to anterior segment anomalies [39-41]. Pathogenic *FOXC1* mutations were identified in PCG cases from southern India [20]. These findings indicate a potential role of the transcription factor *FOXC1* in the development of ocular tissues including the drainage structures. Since neither *CYP1B1* nor *MYOC* could explain the overall genetic contribution to PCG in earlier studies, we have also analyzed *FOXC1*. However, no pathogenic mutations were identified in *MYOC* and *FOXC1* gene in our patients. It, therefore, gives us a premise to assume the non-involvement of both *MYOC* and *FOXC1*.

This difference in mutation spectrum of north Indian and south Indian population may be explained on the basis of different evolutionary history/ethnicity of both populations. India has a heterogeneous population with people from north and south India being ethnically different. The ethno-linguistic composition of the population of India, Pakistan, Bangladesh, Nepal, Bhutan, Maldives, and Sri Lanka mostly falls within two large groups; Dravidian and Indo-Aryan. These groups are further subdivided into numerous subgroups, castes, and tribes. Indo-Aryans form the predominant ethno-linguistic group in Pakistan, India (the central, eastern, western, and northern regions), Nepal, Sri Lanka, and the Maldives. Dravidians form the predominant ethno-linguistic group in southern India and the northern and eastern regions of Sri Lanka (The Indian Genome Variation database [IGVDb]) [42]. The north Indian population is predominantly Aryan population while the south Indian population is Dravidian with totally different morphological phenotype and genetic background. Recently it has been shown that ‘Ancestral North Indians’ (ANI), are genetically close to Middle Easterners, Central Asians, and Europeans, whereas the other, the ‘Ancestral South Indians’ (ASI), is distinct from ANI and East Asians as they are from each other [43]. Thus, though mutations have been identified in *MYOC* and *FOXC1* in PCG cases from south India [16,20], these two genes are not involved in PCG in our patients.

### Conclusion

This is the first study from north India showing non-involvement of *MYOC* and *FOXC1* in the pathogenesis of primary congenital glaucoma. Thus, it is important to screen other loci for involvement in congenital glaucoma in cases which are either negative or heterozygous for *CYP1B1* mutations to have a better insight in disease pathogenesis.
